# Navigating the Emergency Department: A Qualitative Study on Resident Doctors’ Insights Into an Electronic Emergency Department Handbook

**DOI:** 10.7759/cureus.99401

**Published:** 2025-12-16

**Authors:** Udara Wickramanayake, Emelia Brown

**Affiliations:** 1 Emergency Department, Queen Elizabeth Hospital, The Queen Elizabeth Hospital King's Lynn NHS Foundation Trust, Kings Lynn, GBR; 2 Emergency Medicine, Addenbrooke's Hospital, Cambridge University Hospitals NHS Foundation Trust, Cambridge, GBR

**Keywords:** clinical decision tool, digital resources, electronic handbook, emergency medicine resident, healthcare professional development, international medical graduate, medical education technology, medical resident education, qualitative thematic analysis, resident doctor induction

## Abstract

Background

Resident doctors entering Emergency Medicine (EM) face heavy workloads, rapid decision-making demands, and variable access to local protocols. While electronic handbooks show promise in other specialties, evidence from emergency departments (EDs) in the United Kingdom is limited. This study evaluated an electronic ED handbook’s impact on confidence and knowledge among new ED doctors at a District General Hospital.

Methods

Using an interpretivist qualitative design, we conducted semi-structured interviews with purposively sampled 15 resident doctors (Foundation Year 2 (FY2), clinical fellows, general practitioner (GP)/EM trainees), all of whom had used the handbook during induction at Queen Elizabeth Hospital, King’s Lynn (QEHKL), United Kingdom. Interviews (~20 minutes) were conducted by an EM registrar with training in quality improvement and research. These interviews were audio-recorded, transcribed verbatim and pseudonymised, and then analysed using reflexive thematic analysis following Braun and Clarke’s six-phase approach.

Results

Six themes emerged: (i) Orientation and Transition Support - reduced induction overload and clarified expectations, (ii) Rapid Access to Local Protocols and Referral Pathways - single-point access to SDEC, stroke, urology and paediatric pathways, (iii) Decision-Making, Safety and Confidence - bedside guidance for acute coronary syndrome (ACS), head injury, venous thromboembolism (VTE)/deep vein thrombosis (DVT), burns and paediatrics, (iv) Efficiency and Workflow - less time searching intranet, smoother external referrals, (v) Usability and Accessibility - intuitive hyperlinks and mobile use, and (vi) Information Reliability and Maintenance - need for timely updates, fixing broken links and governance. Confidence gains were most pronounced among international medical graduates. All participants recommended the handbook for future cohorts.

Conclusion

The electronic ED handbook appeared to support resident doctors during their transition into EM by enhancing confidence, preparedness, and workflow efficiency. These findings offer useful exploratory insight into how a locally tailored digital tool can assist clinicians early in their ED placement. However, as a qualitative study conducted in a single centre, the results reflect participant perceptions rather than objective outcomes. Future multi-centre or mixed-methods research would be valuable to examine broader applicability and assess measurable effects on practice and patient care.

## Introduction

Practising emergency medicine (EM) in a busy accident and emergency (A&E) department presents unique challenges for resident doctors. According to the 2022 General Medical Council (GMC) National Training Survey in the United Kingdom (UK), EM trainees reported overwhelmingly heavy workloads (77% classified their workload as ‘very heavy’ or ‘heavy’) and a higher burnout risk (32%) compared to other specialities [1]. This underscores the critical need for structured induction processes and accessible support tools to safeguard both performance and wellbeing during transition periods.

Emergency departments (EDs) are often overcrowded, and the majority of patients are acutely unwell. The department requires skilful, knowledgeable doctors to make quick but safe clinical decisions. Resident doctors may suffer from a lack of confidence in making decisions about patient care in a high-pressure environment. This can be worsened by limited senior supervision and inadequate inductions [2]. Existing studies suggest that structured induction tools, whether paper-based booklets or digital decision-support apps, can enhance junior doctor preparedness and confidence. For instance, Thomas et al. found that a resident doctor-designed induction booklet enhanced doctors’ ability to understand rotas, request investigations, and respond to emergencies [3]. Spurring observed that a surgical ‘survival guide’ dramatically increased resident doctor confidence from 41% to over 90% [4]. Furthermore, Dhesi et al. showed that an NHS blood transfusion app improved resident doctors’ clinical decision-making accuracy [5]. However, these interventions were limited by small samples, narrow clinical focus, and reliance on self-reported or simulated outcomes. Importantly, none have evaluated electronic induction tools within EM, where rapid decision-making, high cognitive load, and reliance on numerous local pathways create unique challenges for new doctors. This highlights a clear gap in the literature and reinforces the need to explore how an electronic ED handbook may support resident doctors during their transition into EM. This study aims to fill that gap by examining the impact of an electronic ED handbook on resident doctors’ confidence and knowledge in an A&E department in the UK.

The research question guiding this study is: “How do resident doctors perceive the impact of an electronic ED handbook on their confidence and clinical knowledge during their transition into EM?” This addresses the significant challenges resident doctors face when starting EM rotations, particularly in adapting to the fast-paced and high-pressure environment of A&E departments.

Study objectives

The objectives of this study are to: (i) explore resident doctors’ perceptions of how the electronic ED handbook influenced their confidence during their transition into EM, (ii) examine how resident doctors used the e-handbook to support their understanding of local protocols and clinical systems, and (iii) identify perceived strengths and limitations of the e-handbook as a clinical support and learning tool.

Framework

This study is structured using the PIO (Problem, Intervention, Outcome) framework: (i) Problem: Resident doctors may lack knowledge and confidence when starting in the ED, (ii) Intervention: Implementation of an electronic ED handbook containing protocols, referral pathways, and induction information, and (iii) Outcome: Improved self-reported confidence and knowledge among resident doctors.

## Materials and methods

Development of the electronic handbook 

The development of the electronic ED handbook was informed by both clinical and educational principles. A team of senior consultants and registrars with extensive experience in the ED of Queen Elizabeth Hospital, King’s Lynn (QEHKL), Norfolk, UK, designed the content using current trust, regional, and national protocols to ensure clinical accuracy and alignment with local pathways. The handbook was also shaped by a survey of junior doctors, conducted as part of a Plan-Do-Study-Act (PDSA) quality improvement framework, which identified the specific information new doctors felt they needed during induction. This combination of expert-led content development and user-informed design provided a coherent conceptual basis for the intervention, ensuring that the handbook directly addressed the cognitive load, decision-making demands, and local system challenges faced by new ED doctors. After the electronic handbook was introduced, resident doctors had direct access via their mobile devices and hospital computers. They could utilise this tool to help make real-time clinical decisions on the shop floor.

Research design

This study adopts an interpretivist qualitative research design, chosen for its ability to capture rich, in-depth insights into resident doctors’ experience of utilising the e-handbook. Unlike quantitative research, which relies on numerical measurement and statistical generalisation, qualitative research allows exploration of complex experiences, attitudes, and perceptions that cannot be easily converted to numbers [6]. The incoming cohort of resident doctors to the ED at QEHKL is relatively small, typically comprising 10-15 new doctors every four to six months. For this reason, qualitative research is more suitable, as it often involves smaller sample sizes compared to quantitative research due to data saturation. Data saturation occurs when, after a certain number of responses to the same question, no new information is generated [7-9].

The study is situated within an interpretivist paradigm, which assumes that reality is socially constructed and best understood through individuals’ perspectives [10]. This approach is particularly suitable for exploring how resident doctors experience their transition into the ED, as confidence, decision-making, and perceptions of support are inherently subjective and shaped by personal, social, and organisational contexts. An interpretivist stance allows the research to capture the meanings doctors assign to the electronic handbook, how they integrate it into their workflow, and how it influences their sense of competence and safety in a high-pressure clinical environment. Rather than seeking objective measurement, this paradigm values the depth, nuance, and variation in participants’ lived experiences, making it an appropriate and coherent foundation for this qualitative inquiry.

Literature search strategy

Prior to data collection, a targeted literature search identified existing evidence related to electronic medical handbooks, resident doctor confidence, and EM education and training in the UK. The search (Google Scholar, MEDLINE (Medical Literature Analysis and Retrieval System Online)) used Boolean operators to combine terms such as ‘resident doctor induction’, ‘electronic medical handbooks’, ‘medical apps’, ‘Emergency Medicine training’, and ‘resident doctor confidence’. Literature from the last 10 years was prioritised. UK-based studies were emphasised to ensure contextual relevance; non-UK systems and literature focused on senior clinicians or other professions were excluded. A lack of ED-specific evaluations of electronic handbooks or mobile applications was identified, justifying the present study.

Participant population and recruitment

The study population consists of resident doctors who have recently commenced placements in the ED at QEHKL and who have had access to and used the electronic ED handbook as part of their induction. A purposive, non-probability sampling strategy was used to recruit participants. This sampling approach ensured that participants were specifically selected because they had experience with the handbook, aligning directly with the study objectives.

Recruitment was facilitated by an independent gatekeeper, the Lead for Research, Innovation and Development at QEHKL, who is not part of the ED clinical team, thereby minimising the risk of selection bias. Eligible participants were identified through departmental records, and initial contact was made via an email invitation sent by the gatekeeper. This email explained the purpose of the study and invited participation. Interested individuals were then provided with a Participant Information Sheet (PIS), detailing the study aims, methods, and ethical considerations. Those who agreed to participate were required to complete a written consent form before data collection commenced.

Inclusion criteria were Foundation Year 2 (FY2) doctors, Junior Clinical Fellows, and EM or general practitioner (GP) trainees in an EM rotation who had worked in the QEHKL ED (current or recent) and used the electronic handbook during their placement. Resident doctors who had not worked in the QEHKL ED or had not used the electronic handbook, senior doctors (ST3+ (Stage 3 Specialty Training plus)), nurses, and allied health professionals were excluded.

Ethical considerations

Ethical approval was obtained from the Faculty Research Ethics Panel at Anglia Ruskin University (approval number: ETH2425-6409). Local approval was secured from QEHKL, with the gatekeeper authorised to approach potential participants. Participation was voluntary, with the right to withdraw at any time.

Data collection

Data was collected via semi-structured interviews, conducted face-to-face or virtually, according to participant preference. The interviewer was an EM registrar with training in qualitative research. Each interview lasted approximately 20 minutes and followed a guide exploring confidence, knowledge, and experiences of using the ED handbook in clinical practice. The sample size was 15 participants, reflecting the cohort and anticipated saturation. Of the 15 participants, four were FY2 doctors, four were junior clinical fellows, three were GP trainees, and four were EM trainees (ST1-ST2). Across these groups, six participants were international medical graduates (IMGs). Interviews were audio recorded with participants’ written consent, transcribed verbatim, and pseudonymised to protect confidentiality. Identifiable information was removed, and data were securely stored in password-protected files. In compliance with the Data Protection Act 2018 and UK General Data Protection Regulation (GDPR), all data was processed within the UK. The use of a consistent semi-structured interview guide and deliberate avoidance of leading or confirmatory questions helped minimise the risk of early interviews influencing later data through potential confirmation bias.

Data analysis

We conducted reflexive thematic analysis following Braun and Clarke’s six-phase approach [11]. Transcripts were read repeatedly to support familiarisation, and inductive, data-driven codes were generated manually by the researcher. Codes were then grouped and developed into preliminary themes through iterative comparison and engagement with the data. These themes were reviewed against the full dataset, refined for coherence, and clearly defined to capture patterned meaning across participants’ accounts. The analysis was carried out by an EM registrar familiar with the clinical environment, using reflexive practices to acknowledge how researcher perspective informed interpretation. 

Data saturation was assessed throughout the coding process by monitoring the emergence of new concepts across interviews. Later interviews reiterated the same patterns around improved confidence, rapid access compared with the intranet, clarity of referral pathways, and use of the handbook during time-critical clinical situations. These later interviews provided additional depth but did not generate new codes.

**Figure 1:**
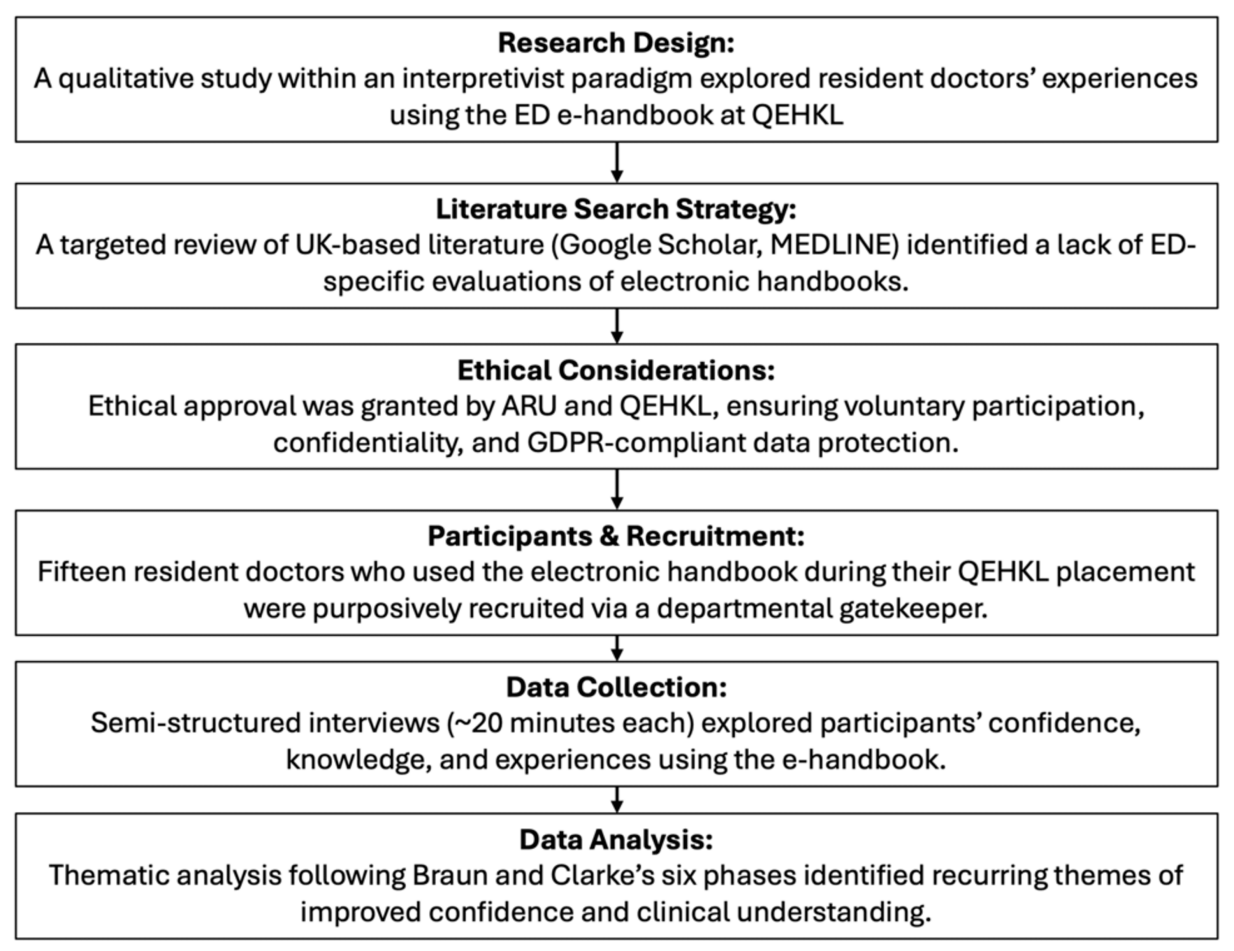
Overview of research methodology QEHKL: Queen Elizabeth Hospital, King’s Lynn; ARU: Anglia Ruskin University; GDPR: General Data Protection Regulation

Figure 1 provides an overview of the study methodology.

## Results

Semi-structured interviews were conducted with the 15 participants. A summary of the recurring responses mapped against interview questions is presented in Table 1, demonstrating how codes repeatedly emerged across participants and confirming that thematic saturation had been achieved.

**Table 1:** Examples of data saturation identified on data analysis IMG: international medical graduate; ACS: acute coronary syndrome; STEMI: ST-segment elevation myocardial infarction; DVT: deep vein thrombosis; VTE: venous thromboembolism

Code	Description	Early Interviews (P1–P10)	Later Interviews (P11–P15)	Saturation Evidence
Improved confidence	Handbook boosted confidence & independence	P1: “Really improved my confidence”; P3: “Improved my competence”; P5: “Boosted my confidence a lot”; P10: “Definitely improved my confidence”	P12, P14, P15 repeated identical points	No new confidence-related insights after P10
Rapid access vs intranet	Faster access, avoids intranet delays	P3: “Found it in seconds… intranet was frustrating”; P9: “Intranet takes ages… handbook has everything readily available”	P13: “Saves time from scruffling through the internet”; P14: “Handy on the phone”	Same comparison repeated; no new information after P9
Referral pathways clarity	Clarity on who to call, when, and how	P4: ”Bleep numbers helped”; P6: “Internal/external contacts”; P8: “Surgical referral pathways”	P11, P12, P15 repeated same points	Pathway clarity saturated by P8
Use in clinical scenarios	Specific cases where handbook guided decisions	P1: CT head, ACS; P2: Burns referral; P5: STEMI; P6: Urology emergencies; P7: DVT	P11: VTE dosing; P9: Polytrauma; P14: ACS	No new clinical categories after P7—same patterns repeated
Induction support	Handbook supplements or replaces incomplete induction	P2: “Much more content than induction”; P3: “Learned more via handbook”; P10: “Induction was generic”	P12: Strengthened induction; P13: Useful because induction was missed	Repeated theme across all interviews
Navigation & usability	Ease of use, hyperlinks, phone access	P2: “Easy to download”; P6: Clear structure; P9: Hyperlinks intuitive	P11, P14, P15 repeated similar comments	No novel usability issues after P9
Limitations & missing content	Broken links, missing guidelines, outdated bleeps	P7: Broken links; P10: Missing psychiatry guidelines	P13: Missing sedation guidelines; P15: Changing bleeps	All limitations relate to missing/outdated content—theme saturated by P10
Help for IMGs	Unique value for international graduates	P3: “Very useful as a new person”; P5: Helped adapt to UK system	P13: IMG section helpful; P14: “Survival guide for me as an IMG”	Repeated across all IMG participants—no new insights later

**Figure 2:**
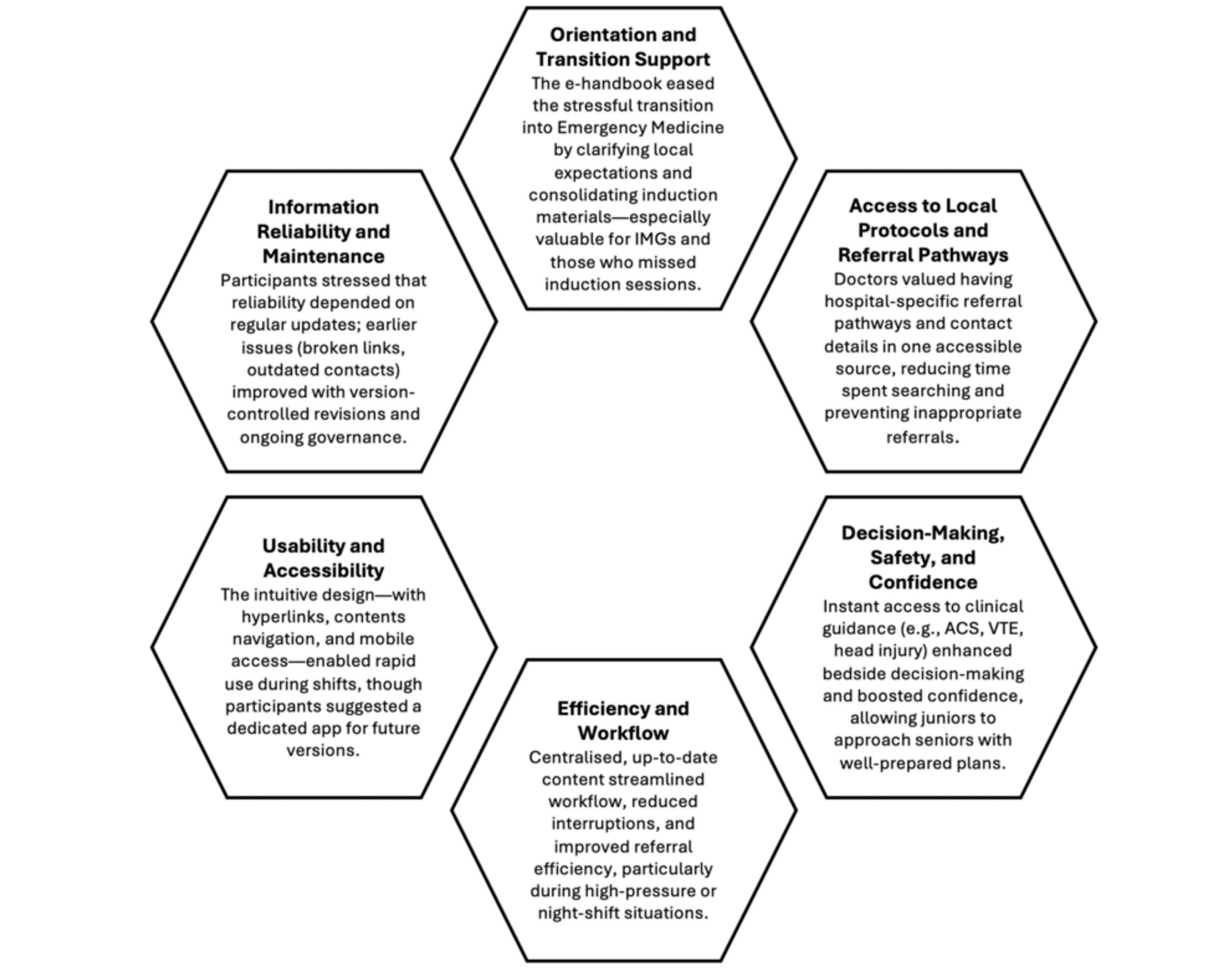
Summary of thematic findings IMG: international medical graduate; ACS: acute coronary syndrome; VTE: venous thromboembolism

Six interviews were virtual and nine face-to-face, according to participant preference and availability. Thematic analysis generated six themes: (i) Orientation and Transition Support, (ii) Rapid Access to Local Protocols and Referral Pathways, (iii) Decision-Making, Safety and Confidence, (iv) Efficiency and Workflow, (v) Usability and Accessibility, and (vi) Information Reliability and Maintenance (Figure 2). Each theme is described in detail, accompanied by participant quotes to illustrate key points. 

Orientation and transition support

Participants consistently described the e-handbook as a vital support during the stressful transition into EM. Beginning work in a new ED, often as the first post in the UK, was experienced as disorientating, particularly in the face of high patient turnover and pressure to perform. The e-handbook reduced this stress by consolidating induction material and clarifying expectations.

*“Initially, it was difficult to navigate referral pathways but after going through the e-handbook, it really helped me to settle in quickly.”* ~P1

For IMGs, the e-handbook was particularly valuable in adapting to unfamiliar systems.

*“This was my very first UK job; the e-handbook helped me a lot during this transition period.”* ~P10

It was also helpful when induction sessions were missed due to absence or sickness.

*“I missed some of the induction… so the e-handbook was quite useful to familiarise myself with local policies and trust guidelines.” *~P8

These findings suggest that the handbook acted as a transitional scaffold, supporting doctors as they navigated unfamiliar systems during their first weeks in the ED. Beyond providing factual information, the resource appeared to reduce psychological stress and early uncertainty, particularly for IMGs, by offering a structured, dependable reference point. This pattern indicates that the value of the intervention lies not only in content provision but in shaping feelings of preparedness and enabling smoother onboarding into a complex clinical environment.

Rapid access to local protocols and referral pathways

A dominant feature was the handbook’s consolidation of hospital-specific protocols and referral processes into a single, accessible resource. Locating referral criteria or up-to-date contact details via the intranet was described as time-consuming and inefficient; the e-handbook streamlined this.

*“Each hospital has their own specialty referral pathways and when you’re new to a hospital it takes time to understand them. The e-handbook has all this information and it’s extremely useful.”* ~P2

*“I went to look into the DVT (deep vein thrombosis) guideline and found it within seconds. It gave me all the answers I need.”* ~P3

Clarity and accessibility of referral information were considered essential for safe decision-making and avoiding inappropriate escalation. Examples included same-day emergency care (SDEC), stroke, urology, and paediatric pathways.

*“Probably the most helpful content for me was surgical referrals. It had information on who is on call and whom I should be referring to during out-of-hours. For example, if I had a urology referral, I would go straight to the urology registrar during 8 to 5 pm, but after 5 o'clock I would go to the general surgical team.”* ~P6

The repeated emphasis on speed and clarity reflects the significant cognitive effort required to locate dispersed guidelines within ED workflows. Participants contrasted the handbook with the intranet, implying that the tool reduced extraneous cognitive load, enabling quicker and safer decisions. This suggests that access to consolidated local information is not merely convenient but can also be beneficial for maintaining efficient patient flow and reducing risk in time-critical situations.

Decision-making, safety, and confidence

Participants reported that the e-handbook directly supported safe clinical decision-making at the bedside for common presentations such as acute coronary syndrome (ACS), head injury, venous thromboembolism (VTE), burns, and paediatric illnesses.

*“Head injury…By taking a quick look at the e-handbook, we can decide to do the CT head scan or not. It helps us to make quick decisions in the ED.”* ~P1

*“The VTE page is a very useful guideline to help with prescribing, without having to scruffle through the intranet.”* ~P7

Doctors used the handbook to prepare management plans before seeking senior advice.

*“Before you discuss with a senior, you are more confident and prepared to present the case and your management plan.” *~P14

Confidence gains were particularly valuable for IMGs new to the NHS.

*“My confidence was quite low when I started, but after using the handbook, it boosted my confidence a lot.”* ~P5, an IMG in their first NHS job

Participants’ accounts indicate that the handbook served as a form of pre-escalation validation, allowing clinicians to check their reasoning prior to seeking senior input. This pattern reflects an important developmental function: supporting junior clinicians to bridge the gap between theory and real-time clinical judgement. For IMGs, the tool appeared to mitigate uncertainty arising from unfamiliarity with UK pathways, reinforcing the role of localised digital guidance in building safe, autonomous decision-making practices.

Efficiency and workflow

The handbook was perceived to enhance efficiency by centralising information, thereby reducing delays from intranet searches or repeated interruptions, especially during night shifts and urgent situations.

*“Saved my time a lot… everything that I need to know… summarised in a very nice organised way.”* ~P9

*“The e-handbook helps to make quick decisions without having to go to the ED consultant all the time.”* ~P2

Participants reported that the handbook provided up-to-date guidance on external tertiary hospital referrals, including clear indications for transfer, appropriate timing, the relevant specialty contacts, and the essential information required to accompany the referral.

*“Polytrauma… I had to consult cardiothoracic surgeons… I just opened the e-handbook and found everything I need… managed to complete the referral within a reasonable time.”* ~P9

The perceived efficiency gains described by participants go beyond simple time savings and extend into workflow optimisation. The handbook appeared to streamline information-seeking behaviour, which could reduce interruptions to seniors and prevent delays caused by navigating the intranet. This suggests that digital point-of-care resources may contribute meaningfully to ED operational efficiency by supporting rapid task-switching and reducing bottlenecks in clinical decisions.

Usability and accessibility

Overall, the handbook was described as intuitive, navigable, and well-suited to the fast-paced clinical environment. Hyperlinks and a contents page enabled rapid movement between topics. Because it was stored on their personal phones, the e-handbook was described as a readily available support tool that accompanied them throughout their shifts.

*“The hyperlink system is very easy to navigate… Just go to the contents page and click on the link.” *~P5

*“The e-handbook is in your phone and always there if you need support.”* ~P10

However, others noted that accessibility could be further improved, particularly through the development of a dedicated app.

*“Creating a more advanced app would be more useful.”* ~P8

The strong emphasis on portability, intuitive navigation, and mobile-based access highlights that usability was central to real-world adoption. Participants’ descriptions of the handbook as something they “always had with them” illustrate how deeply it became embedded in daily workflow. This indicates that digital tools designed for acute care settings must prioritise instant availability to gain traction in a high-pressure environment where clinicians often cannot afford delays.

Information reliability and maintenance

Reliability was seen as dependent on accuracy and completeness. Reported issues included broken links, outdated contact numbers, and missing content in earlier versions.

*“There were a few links that were not working.” *~P1

*“Rapidly changing bleep numbers need to be updated.”* ~P9

Participants emphasised the need for regular review and updating to maintain trust.

*“Updating the handbook frequently with new policies and changes is important.”* ~P12

Encouragingly, some noted that subsequent editions had addressed gaps.

*“The first edition… had a lack of guidelines… which has been updated in subsequent editions.”* ~P7

Concerns regarding broken links and outdated contact details underscore the importance of maintaining trust in the reliability of digital clinical tools. Inconsistent or outdated information risks undermining confidence in the resource, especially in high-stakes ED scenarios. These reflections highlight the need for active governance, version control, and continuous updates to ensure that digital handbooks remain credible and safe for clinical use.

Summary of results

Resident doctors experienced the electronic ED handbook as a highly valuable support during their transition into EM. It reduced induction overload, centralised access to local pathways, enhanced clinical decision-making, improved workflow efficiency, and supported confidence and autonomy. The resource was particularly transformative for IMGs, who found it helpful for adapting to the UK system and developing independent clinical confidence.

Importantly, links to patient safety could be inferred from the data, as interviewees reported that the handbook helped reduce inappropriate referrals, minimise delays, and ensure safe escalation. It also reshaped interactions with senior colleagues, allowing juniors to present well-prepared management plans and reducing unnecessary interruptions. The e-handbook has undergone three versions, developed as a version-controlled quality improvement project led by ED senior registrars and supervised by consultants. Updated editions are distributed to current and incoming resident doctors, with a lecture delivered during departmental teaching on navigation, content, and updates. Feedback from resident doctors is gathered after one to two months of use, allowing the team to address gaps and keep each edition responsive to evolving clinical needs, ensuring the handbook remains a dynamic and sustainable tool.

## Discussion

Overall, participants described the e-handbook as a useful support tool that eased transition into the ED, reduced stress, clarified referral pathways, and provided real-time access to hospital-specific protocols. It has reportedly enhanced clinical confidence, improved efficiency, and reduced dependence on senior colleagues. The value was particularly emphasised by IMGs, who highlighted its role in adapting to unfamiliar systems. Limitations identified included the need for timely updates, governance of content, and enhanced accessibility by developing the e-handbook into an advanced mobile app.

Interpretation of findings in relation to educational and social theories

Vygotsky’s Zone of Proximal Development and scaffolding

The e-handbook could be seen to function as a scaffold, enabling resident doctors to perform beyond their independent level of competence during the first few months, consistent with Vygotsky’s Zone of Proximal Development [12]. By providing protocols, referral pathways, and structured clinical decision-making tools, it allowed doctors to attempt tasks they could not yet achieve unaided. For example, participants reported being able to prepare clinical management plans for senior review, which fostered confidence and a sense of readiness. This reflects the process of scaffolding, whereby external supports are gradually removed as learners gain competence. Over time, repeated reference to the handbook helped participants feel they were less dependent on seniors, building autonomy and accelerating professional growth.

Dreyfus Model of Skill Acquisition

Participants’ experiences align with the early stages of the Dreyfus model of skill acquisition [13]. Analysis of the data suggests that the e-handbook supported the transition from novice, reliant on strict rules, to advanced beginner, capable of applying knowledge in context. By offering structured frameworks for safe decision-making, the handbook helped resident doctors recognise patterns, respond more independently, and develop clinical judgement. This progression echoes expectations outlined in the General Medical Council’s (GMC's) Generic Professional Capabilities framework [14] and the Royal College of Emergency Medicine (RCEM) curriculum [15], which stress not only technical skills but also safe practice, teamwork, and decision-making under pressure.

Bloom’s Taxonomy and Dale’s Cone of Learning

Several participants described induction lectures as overwhelming, with much content quickly forgotten, whereas the e-handbook was perceived as concise, relevant, and reusable. This supports Bloom’s taxonomy [16], which highlights the need for learners to progress from recall to application. Similarly, Dale’s cone of learning suggests that active engagement during real-world problem-solving results in greater knowledge retention compared to passive lecture-based formats [17]. By reducing information overload and allowing quick access to essential guidance, the e-handbook likely reduced cognitive load, freeing capacity for clinical reasoning. This may also contribute to lowering stress and burnout in a specialty known for its intensity.

Cognitive load, stress, and burnout

EM is characterised by high cognitive demands. The e-handbook reportedly reduced information overload by centralising key resources and streamlining access, thereby lowering extraneous cognitive load. This allowed resident doctors to devote more mental energy to clinical reasoning and patient interaction. Importantly, this reduction in unnecessary stressors may contribute to mitigating burnout, a recognised risk in EM trainees [18].

Integration with RCEM and GMC standards

Taken together, these findings highlight the e-handbook as a potential bridge between formal curriculum requirements and real-world practice. The RCEM curriculum emphasises rapid decision-making, local knowledge, and multidisciplinary collaboration [15], while the GMC’s professional standards stress safety, quality improvement, and professional judgement [14]. The handbook operationalised these expectations by providing an accessible, evidence-based resource embedded within daily workflow. In doing so, it not only supported experiential learning but also strengthened alignment between policy frameworks and clinical reality.

Strengths and limitations

Strengths

A major strength of this study was the inclusion of a diverse participant group. FT2 doctors, junior clinical fellows, GP trainees, EM trainees, and IMGs all contributed, ensuring a wide range of perspectives. This diversity enriched the findings and highlighted how the e-handbook addressed challenges across different levels of training and backgrounds.

The use of semi-structured interviews added further strength, allowing participants to discuss both practical experiences and emotional aspects of transition. This flexibility generated insights that might not have emerged through surveys or structured approaches. For IMGs, in particular, the interviews captured the handbook’s role in fostering confidence and integration into the NHS.

Thematic analysis, guided by Braun and Clarke’s framework, ensured a systematic approach to coding and theme development. The integration of participant accounts with educational and social theories strengthened the interpretation, situating findings within established models of learning and competence development.

Finally, the study addressed a gap in the literature by focusing on the use of an electronic handbook in a UK Emergency Department. Given the unique demands of ED practice, high turnover, unpredictable workloads, and reliance on local referral pathways, this adds originality and practical relevance.

Limitations

The study was conducted in a single district general hospital, which limits generalisability. Larger teaching hospitals and major trauma centres may face different challenges and employ alternative induction methods. The small sample size, while appropriate for qualitative research and limiting data saturation, also restricted the breadth of perspectives.

A further limitation of this study is its reliance on self-reported perceptions without triangulation through observational data, document review, or objective usage metrics. While participants consistently described the handbook as valuable, these accounts cannot be independently verified and do not provide objective evidence of outcomes such as reduced errors, faster decision-making, or improved patient flow. As the findings reflect participants’ experiences rather than measured behavioural change, future mixed-methods studies incorporating observational or quantitative data would strengthen the validity and generalisability of these conclusions.

Furthermore, the researcher’s position should be considered when reviewing the findings. The analysis was conducted by an EM registrar familiar with the clinical environment and with prior experience using the e-handbook. While this insider perspective supported contextual understanding of the data, it also carries the potential for confirmation bias, particularly in interpreting themes that aligned with the researcher’s expectations or professional experience. To mitigate this, reflexive practices were used throughout analysis, including regular revisiting of codes, comparison across transcripts, and deliberate consideration of disconfirming or critical participant accounts. 

Despite these limitations, the study still provides meaningful insights and lays a foundation for future multi-centre and mixed-methods research to explore both experiential and measurable outcomes.

## Conclusions

This study indicates that an electronic ED handbook can support resident doctors during their transition into EM by enhancing confidence, preparedness, and workflow. In particular, IMGs found the resource transformative in adapting to UK practice. However, these findings are based on subjective participant accounts from a small, single-site qualitative study, and therefore reflect perceptions rather than objectively measured outcomes such as changes in patient care or error rates. The results should be interpreted within this methodological context, recognising that experiences may vary in other settings. Nevertheless, the study offers valuable insight into how locally tailored digital tools can assist clinicians during the early stages of ED practice and highlights the need for future multi-centre or mixed-methods research to examine broader applicability and objective impact.

Recommendations for future research could include conducting larger, multi-centred evaluations to test transferability and assessing objective outcomes such as medical error rates and decision-making times. Mixed-methods designs would provide a fuller picture of the impact of an ED electronic handbook. Following this, further recommendations could then be considered for policy and practice.
